# Frequency-Domain Method for Characterization of Upconversion Luminescence Kinetics

**DOI:** 10.1021/acs.jpclett.3c00269

**Published:** 2023-04-03

**Authors:** Lucía Labrador-Páez, Jouko Kankare, Iko Hyppänen, Tero Soukka, Elina Andresen, Ute Resch-Genger, Jerker Widengren, Haichun Liu

**Affiliations:** † Department of Applied Physics, KTH Royal Institute of Technology, SE-10691 Stockholm, Sweden; ‡ Department of Chemistry, University of Turku, FI-20014 Turku, Finland; § Department of Life Technologies/Biotechnology, University of Turku, FI-20520 Turku, Finland; ∥ Division of Biophotonics, Federal Institute for Materials Research and Testing (BAM), Richard-Willstätter-Str. 11, 12489 Berlin, Germany

## Abstract

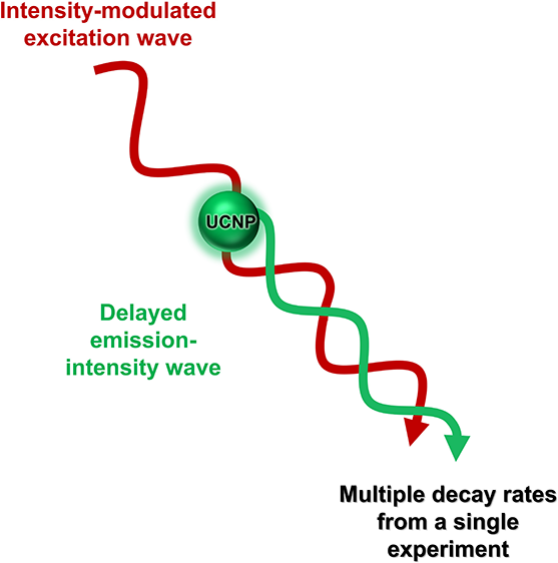

The frequency-domain (FD) method provides an alternative to the commonly used time-domain (TD) approach in characterizing the luminescence kinetics of luminophores, with its own strengths, e.g., the capability to decouple multiple lifetime components with higher reliability and accuracy. While extensively explored for characterizing luminophores with down-shifted emission, this method has not been investigated for studying nonlinear luminescent materials such as lanthanide-doped upconversion nanoparticles (UCNPs), featuring more complicated kinetics. In this work, employing a simplified rate-equation model representing a standard two-photon energy-transfer upconversion process, we thoroughly analyzed the response of the luminescence of UCNPs in the FD method. We found that the FD method can potentially obtain from a single experiment the effective decay rates of three critical energy states of the sensitizer/activator ions involved in the upconversion process. The validity of the FD method is demonstrated by experimental data, agreeing reasonably well with the results obtained by TD methods.

Lifetime (or its inverse, decay rate) is a very important photophysical parameter characterizing luminescent materials and has important applications. For instance, this parameter can be exploited for the purpose of chemical sensing and imaging, as it is environment-sensitive while less prone to artifacts than other readouts such as (relative) luminescence intensities or intensity ratios.^1−3^ In particular, luminescence lifetime imaging is now used in clinical practice for noninvasive sensing and imaging.^4,5^ Therefore, it is valuable to develop simple techniques for assessing the decay rates of luminescent labels with low-cost instrumentation.

Among luminescent labels, lanthanide-doped luminescent nanoparticles, both downshifting and upconversion nanoparticles (UCNPs), stand out due to their merits, including excellent photostability, large energy gap between excitation and emission wavelengths, and emission in a multitude of characteristic narrow emission bands. These bands cover a very broad spectral range, ranging from ultraviolet (UV), visible (VIS), and near-infrared (NIR), to short-wave infrared (SWIR).^6−8^ The decay rates of the energy states of lanthanide dopants can be engineered by tailoring the doping level or the structure of the nanoparticles. These strategies have allowed advances in applications of lanthanide-doped luminescent nanoparticles in fields such as nanomedicine, biosensing, clinical diagnostics, super-resolution microscopy, and anticounterfeiting technology.^9−16^

The most widely used approach to measure luminescence lifetime of luminescent materials is the time-domain (TD) method, where the material is excited by a short laser pulse and the evolution of its emission intensity is observed and fitted with (multi)exponential decay(s) to extract the decay rate(s). The determination of decay rates of lanthanide-doped luminescent nanoparticles with down-shifted luminescence is straightforward using the TD method, although it might not always be trivial to fit the emission decay profile with (multi)exponential decay(s) and to decouple (multiple) decay components (Figure 1a). However, for nonlinear systems with upconverted emission such as lanthanide UCNPs (Figure 1b), the extraction and interpretation of the decay rates is more complex. This is because of their more complicated, excitation intensity- and history-dependent luminescence kinetics, featuring typical rise and decay profiles, and involving multiple energy states and energy transfer processes.^17,18^ In the most prominent energy transfer upconversion (ETU) processes, as exemplified by a two-photon upconversion process in Figure 1b, an ion acting as sensitizer absorbs photons of low energy and transfers them consecutively to another ion, the activator, so that it gets excited and emits higher-energy photons. Generally, upconversion luminescence (UCL) of UCNPs exhibits a complicated excitation impulse response function (IRF), which depends not only on the decay rate of the emitting state of the activator ion but also on the decay rate of the excited state of the sensitizer ion and that of the intermediate state of the activator ion, as well as the energy transfer rates between the sensitizer and the activator. Further, the observed UCL kinetics represents a collective response of the network of numerous sensitizer and activators ions within individual nanoparticles.^13,19^ For these reasons, the TD method can often not decode the rise and decay kinetics of UCNPs and also not obtain all decay rate components. The possible dependence of UCL kinetics on excitation intensity and pulse width makes this task even more challenging.^20,21^

**Figure 1:**
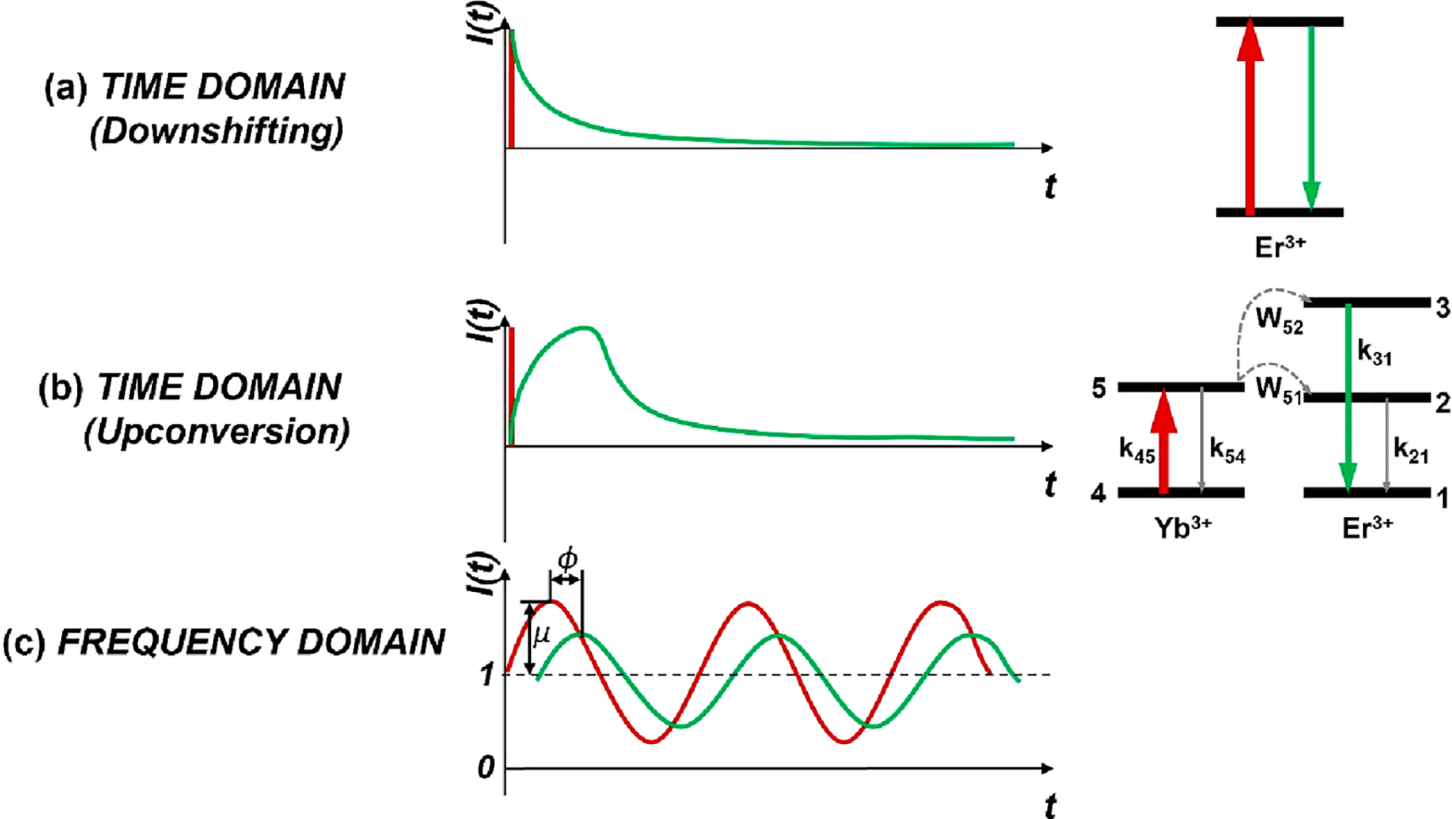
(a) Left: Schematic representation of the evolution of luminescence intensity in the time-domain method, for lifetime measurements of down-shifted emission. Right: Schematic representation of the energy level diagram of a downshifting system. (b) Left: Schematic representation of the evolution of luminescence intensity in the time-domain method for lifetime measurements of upconverted emission. Right: Schematic representation of the energy level diagram of an upconversion system, consisting of sensitizer and activator ions. The transitions of interest for the rate-equations-based model and their corresponding decay rates are shown (red for excitation, green for the two-photon upconversion emission under study, dashed for energy transfer). Herein, *k*_45_ represents the pumping rate of the sensitizer ground state, *k*_54_ the decay rate of the sensitizer excited state, *k*_21_ the decay rate of the activator intermediate state, *k*_31_ the decay rate of the activator UCL emitting state, and *W*_51_ and *W*_52_ the energy transfer coefficients from the sensitizer excited state to the activator intermediate and UCL emitting state, respectively. (c) Schematic representation of the evolution of luminescence intensity in the frequency-domain method for lifetime measurements of both down-shifted and upconverted emission (red for the excitation, green for the emission, μ for the modulation amplitude, and ϕ for the phase lag).

Alternatively, decay rates can be quantified using the frequency-domain (FD) method, where the luminescent material is excited by an intensity-modulated excitation wave with a certain frequency (Figure 1c). Due to the finite decay rate of the involved energy states, the modulated emission is delayed in time and demodulated in amplitude with respect to the excitation wave, by a factor that depends on the magnitude of the excited-state decay rate relative to the employed modulation frequency.^22^ The luminescence decay rate can then be extracted from the phase lag and the demodulation factor of the emission wave. Multiple reviews have compared the TD and FD methods.^2,22−24^ The FD method has advantages when multiple decay rates are to be determined with high reliability and accuracy, because the analysis is based on separating bell-shaped curves. Such curves that overlap are more easily distinguishable than corresponding exponential decays (in the TD method).^25^ For that reason, and especially with instrumental noise present, the FD method is preferred for the analysis of multilevel systems, where each level could impose its specific effect on the overall luminescence kinetics.^26−29^ Particularly for lanthanide-doped luminescent materials, the generally long lifetimes of their excited states allow measurements to be performed in a low frequency range, further reducing the requirements on the instrumentation.^2,29,30^

Although the FD method has been thoroughly developed and extensively used for characterizing luminescent materials with a linear response to excitation intensity, its theory has not been adequately developed for characterizing nonlinear luminescent materials such as lanthanide UCNPs. In the past, the applications of the FD method on UCNPs inherited the linear models applicable to lanthanide complexes and ignored the nonlinearity of UCNPs.^13,28−31^ Thus, the data interpretation and the assignments of returned decay rates remain unsatisfactory. In this work, by using a simplified rate-equation model for a standard two-photon energy transfer upconversion process, we in theory thoroughly analyzed the response of UCNPs to sinusoidal-wave excitation in the FD approach. We found that the FD method can potentially extract the effective decay rates of three critical energy states of the sensitizer and activator ions involved in the upconversion process, from a single experiment. This was validated with experimental data and benchmarked with the results obtained by TD methods.

In the analysis of the response of UCNPs to a sinusoidally modulated excitation intensity in the FD method, we consider the most representative two-photon energy transfer upconversion process shown in Figure 1b. Here, the sensitizer ion has a two-energy-level structure, as the Yb^3+^ ion, the most common sensitizer, while the activator ion has a simplified three-level structure. In the upconversion emission process, the activator ion at the ground state (state **1**) is first excited to state **2** through a phonon-assisted energy transfer from an excited sensitizer ion, and further excited to state **3** through a second energy transfer process. Subsequently, the UCL is generated by transition from state **3** to state **1** of the activator ion. Our previous theoretical analysis showed that this simplified mechanism can well represent the schemes for several well-known upconversion emission bands under Yb^3+^ sensitization upon ∼980 nm excitation, i.e., the green emissions of Er^3+^ ions (^2^H_11/2_/^4^S_3/2_ → ^4^I_15/2_) and Ho^3+^ ions (^5^S_2_/^5^F_4_ → ^5^I_8_), and the NIR emission of Tm^3+^ ions (^3^H_4_ → ^3^H_6_), all originating from a two-photon process.^32^ The UCL kinetics of this system can be modeled using the following set of differential equations:
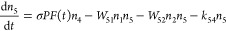
1
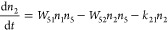
2
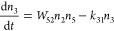
3where *n*_*i*_ is the population density of state ***i***, *k*_*ij*_ the decay rate from state ***i*** to state ***j***, σ is the absorption cross-section of Yb^3+^ ions at ∼980 nm, *P* is the excitation photon flux, *W*_*ij*_ the energy transfer rate from state ***i*** to state ***j***, and *F*(*t*) the modulation function of the excitation intensity. It is assumed that the population densities of the ground states, i.e., state **1** and state **4**, are constants (assuming that only a very minor fraction of the donor and acceptor ions are at an excited state). By defining
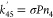
4
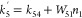
5
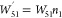
6and ignoring the term *W*_52_*n*_2_*n*_5_ in eq 1 (because *n*_2_ ≪ *n*_1_ is generally valid under mild excitation and *W*_51_ and *W*_52_ can be considered similar in magnitude), eqs 1 and 2 can be rewritten as
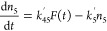
7
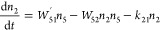
8Equations 3, 7, and 8 comprise the model adopting Approximation I (App I), which is essentially corresponding to medium excitation, i.e., under which the contributions of the radiative decay (*k*_21_*n*_2_) and the ETU process (*W*_52_*n*_2_*n*_5_) to the depopulation of state **2** are comparable. Under a sinusoidal excitation with a circular frequency ω and a modulation amplitude μ, i.e.,
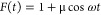
9the amplitude of the UCL signal, *S*(ω), proportional to *n*_3_, to be experimentally recorded on a dual-phase lock-in amplifier, can be given by (see Section 1 in the SI for details):
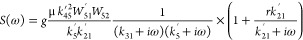
10Here
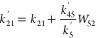
11representing the observed combined decay rate of state **2** and
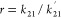
12representing the fraction of the radiative decay rate by the transition state **2** → state **1** in the overall depopulation rate of state **2**. Here, *g* represents the instrument gain factor accounting for the luminescence signal detection efficiency and *i* the imaginary unit.

Using the conventions described in reference (29), different terms in eq 10 can be separated, resulting in
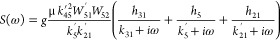
13where
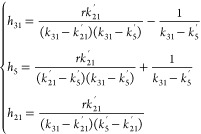
14In experiments, a dual-phase lock-in amplifier can record the real part of the amplitude of the emission as the in-phase signal, *S*_*x*_(ω), and its imaginary part as the out-of-phase (or quadrature) signal, *S*_*y*_(ω), i.e.,
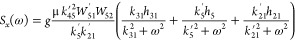
15
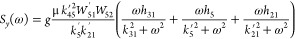
16The in-phase and quadrature signals are connected by the Kramers–Kronig relation, i.e., all the information on the frequency response of the system can be obtained from just one of them.^29^

Alternatively, the lock-in output from eq 13 can be expressed in the following form (see Section 1 in the SI for details):
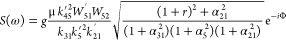
17with α_31_ = ω/*k*_31_, α_5_ = ω/*k*_5_^′^, α_21_ = ω/*k*_21_^′^, and Φ the overall phase lag of the UCL signal relative to the excitation wave, which is given by
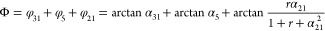
18According to eq 18, the overall phase lag of the UCL is not only dependent on the decay rate of the emitting state (*k*_31_) but also on those of another two important intermediate energy states involved in the upconversion process, affecting the sensitizer excited state (*k*_5_^′^) and the intermediate state of the activator (*k*_21_^′^). This is in line with our previous qualitative analysis on the influential factors of the phase lag of UCL using a simplified phenomenological two-term IRF for the UCL^33^ but provides a high accuracy. It is also predicted that the phase lag of UCL can easily exceed 90° because of the additive effect of the phase lags associated with each of the involved energy states, given that the linear emission from an excited state typically exhibits a phase lag in the range of 0–90° dependent on the modulation frequency.^22^ Experimentally, the phase lag as a function of modulation frequency can be either recorded directly (depending on the instrument) or calculated as an inverse tangent of the ratio of the quadrature and in-phase signals.

An additional approximation, App II, was also considered, where the nonlinear component *W*_52_*n*_2_*n*_5_ in eq 8 was also neglected. This treatment is reasonable when the linear decay term (*k*_21_*n*_2_) is the dominating (actually sole) depopulation channel of state **2**, valid under weak-excitation condition.^32,34^ The difference between these two approximations can be explicitly expressed with a parameter *r*, which attains the value 0 < *r* < 1 in App I (cf. eqs 12 and S31′) and *r* = 1 in App II.

The fitting of experimentally recorded in-phase or quadrature data to eq 15 or 16 with a proper constraint on the *h*-coefficients (equation S55′) will yield the three critical decay rates of the upconversion luminescence process, i.e., *k*_31_, *k*_5_^′^, and *k*_21_^′^. Note that *k*_5_^′^ represents an effective decay rate of state **5** that groups all the depopulation processes affecting state **5** according to eq 5 and that *k*_31_ and *k*_5_^′^ would be independent of excitation intensity but *k*_21_^′^ dependent according to eqs 4 and 11. The fitting procedure is discussed in detail in Section I in the SI.

To test the validity of the FD method in characterizing luminescence kinetics of UCNPs, we first did FD measurements on a dispersion of Yb^3+^-Er^3+^ codoped NaYF_4_ nanoparticles in oleic acid (OA) using sinusoidally modulated excitation at 975 nm. We then analyzed the response of the emission of Er^3+^ ions from the ^4^S_3/2_ → ^4^I_15/2_ transition (543 nm). The nanoparticles studied were NaYF_4_:17%Yb,3%Er UCNPs with an average diameter of 27.2 (±1.5) nm (Figure S2). Using the equations derived from App I, the quadrature data can be well fitted to eq 16, as shown in Figure 2a. As seen, the quadrature signal consists of an overlap of multiple basis curves, the central frequencies of which are those of the decay rates controlling the kinetics of the UCL. The values of the three decay rates are returned as *k*_31_ = 18048 ± 174 s^–1^, *k*_5_^′^ = 6000 ± 30 s^–1^, and *k*_21_^′^ = 450 ± 20 s^–1^. The Kramers–Kronig transform of the quadrature data to in-phase data is presented in Figure 2b. The good quality of this fit justifies the simultaneous fit of the real and imaginary data as an Argand diagram (Figure 2c), returning very similar decay rate values. Figure 2d presents the experimental phase lag data of the same emission band of the sample. As can be seen, the phase increases with increasing the frequency and can easily exceed 90° and approach 180°, in line with our previous report,^13^ supporting the above theoretical analysis on the phase lag.

**Figure 2:**
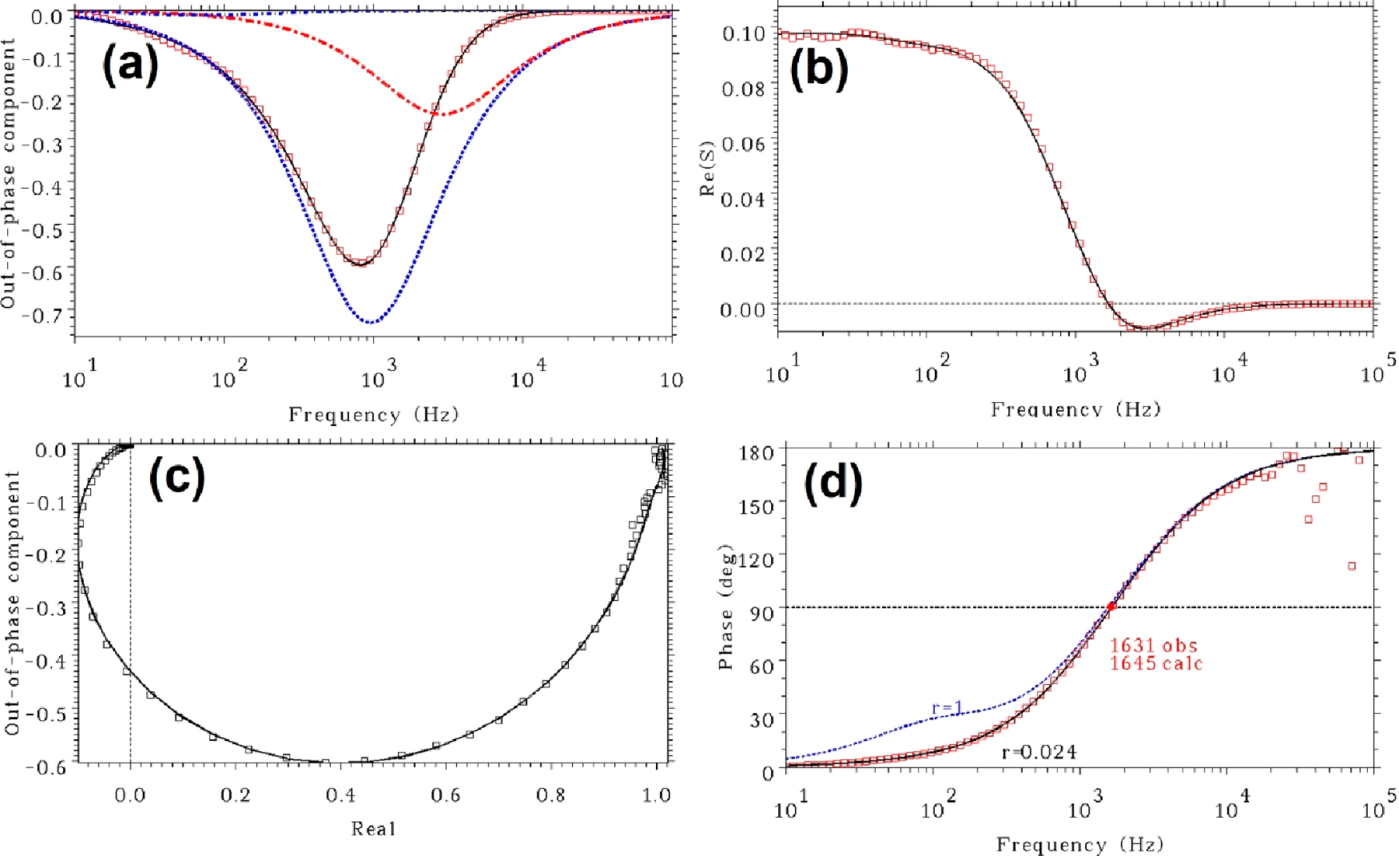
Frequency-domain characterization of the 543 emission of NaYF_4_:Yb,Er upconversion nanoparticles in oleic acid under sinusoidal excitation at 975 nm with the average excitation intensity of 110 W/cm^2^ and modulation degree μ = 0.1. Note that the modulation degree μ is dimensionless according to the definition in eq 9, and the absolute excitation intensity has been taken into account in the parameter *k*_45_^′^. (a) Quadrature data fitting (red squares for experimental data, black solid line for fitting to eq 16, and dashed lines for different basis components with the red dashed line having a positive sign). (b) Kramers–Kronig transform of the quadrature data to in-phase data (red squares for experimental in-phase data, black solid line calculated from experimental quadrature data). (c) Argand diagram of data (black squares) and fitting (black solid line). The fitting was done simultaneously to eqs 15 and 16. (d) Phase lag data fitting to eq 18. Red squares are experimental data corresponding to the inverse tangent of the ratio of quadrature and in-phase signals, the black solid line for fitted data of App I, and blue dashed line for fitted data of App II. *r* = 1 represents App II, *r* = 0.024 for App I. Notations 1631 and 1645 refer to the experimental and calculated frequencies where the phase lag is 90°.

Using the above returned decay rates as constants, the phase lag data can be well fitted to eq 18 using *r* as an adjustable parameter (Figure 2d), yielding *r* = 0.024. *k*_21_ was then determined to be *k*_21_ = *rk*_21_^′^ ≈ 11 s^–1^. Figure 2d also shows the fitting result of App II (blue dashed line, marked by *r* = 1). The misfit is very significant almost for all frequencies below 2000 Hz. The comparison between the fitting qualities of the two approximations shows the validity of App I over App II. This reveals that under this measurement condition (average excitation intensity 110 W/cm^2^) the escape process of state **2** was not dominated by the linear decay process, indicating relatively strong excitation. In addition to the value of *r*, Figure 2d includes the experimental and calculated values of frequencies where the phase lag attains the value 90°.

In order to examine the reliability of the results obtained by the FD method, we also measured the decay rates of the same NaYF_4_:Er,Yb UCNPs in OA using the TD method under direct excitation. To obtain *k*_5_^′^, the nanoparticles were excited by a pulsed 978 nm laser with a pulse width of 20 μs, and the luminescence decay curve at 1010 nm (the Yb^3+^ ions emission) was recorded (Figure 3a). The effective decay rate (*k*_eff_) was evaluated from the emission intensity decay profile, *I*(*t*), by^35^
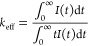
19*k*_5_^′^ was determined to be ∼6140 s^–1^ using the data points in the range of 0–1600 μs. Note the intensity decay of the Yb^3+^ 1010 nm emission can also be well fitted with a single exponential function (the black solid line in Figure 3a), returning a similar decay rate value. In the case of *k*_31_, the excitation wavelength was shifted to 485 nm, so that it was also a down-shifted emission process, opening a possibility to obtain the more intrinsic decay rate of the emitting energy level, ^4^S_3/2_. However, it should be noted that the emission intensity decay profile at 543 nm apparently presents more than one exponential decay component (Figure 3b). This reflects the complexity of luminescence kinetics of lanthanide-doped nanoparticles even under direct excitation,^19^ fundamentally originating from the complex energy-transfer interactions between the lanthanide dopants (e.g., energy transfer from the Er^3+^^4^S_3/2_ state to the Yb^3+^ ground state^37^ in the current case). *k*_31_ was estimated to be ∼14443 s^–1^ using the data in the range of 0–300 μs according to eq 19. Due to the limitations of the available equipment to detect the emission of the Er^3+^^4^I_11/2_ → ^4^I_15/2_ transition, we could not experimentally determine *k*_21_ using the TD method under direct excitation.

**Figure 3:**
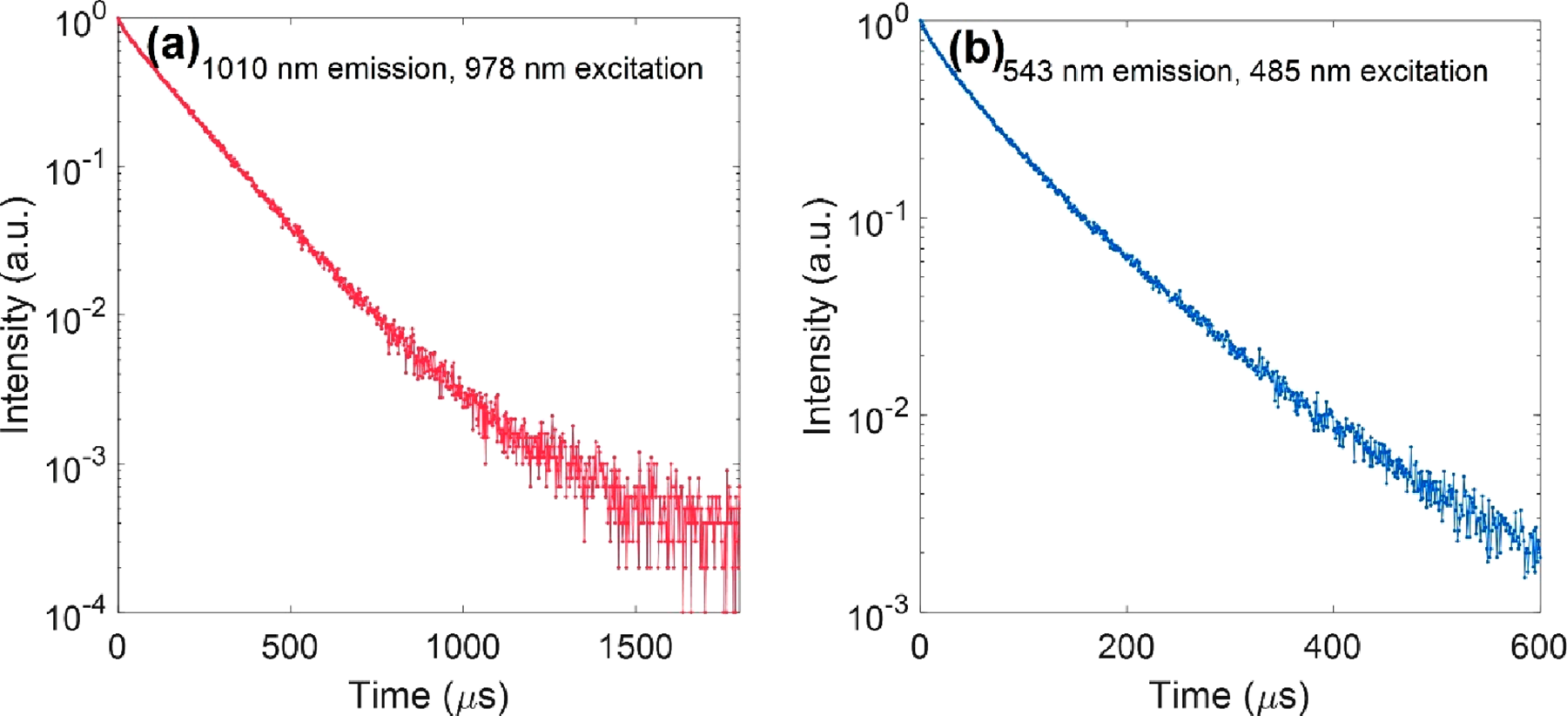
Intensity decay in the time domain method of NaYF_4_:Yb,Er upconversion nanoparticles in oleic acid under direct excitation, detecting their emissions at (a) 1010 nm (from ^2^F_5/2_ energy level of Yb^3+^ ions) under excitation at 978 nm (pulse width 20 μs) and (b) at 543 nm (from ^4^S_3/2_ energy level of Er^3+^ ions) under excitation at 485 nm (pulse width 10 μs), respectively.

We also did measurements on the luminescence decay of the same UCNPs under multiphotonic excitation, the most widely used approach to characterize UCL kinetics. The decay of the green emission of Er^3+^ ions at 543 nm was measured under pulsed excitation at 978 nm with a pulse width of 20 μs. As shown in Figure 4, the emission intensity evolution shows a typical complex kinetics of UCNPs, featuring both a rise and a decay process. Theoretical analysis in our previous work revealed that the time evolution of upconversion luminescence following a short-pulse excitation can convolve the effects of multiple rate constants.^19,33^ Specifically, for a standard two-photon upconversion luminescence generated by two successive energy transfer steps from the sensitizer to the activator (Figure 1b), the emission rise-decay profile right after the termination of the short excitation pulse can contain multiple exponential components characterized by, e.g., rate constants *k*_31_, 2*k*_5_^′^, and *k*_5_^′^ + *k*_21_.^19,33^ The rise-decay profile of the green emission of Er^3+^ ions at 543 nm was fitted using equation S59′ (see Section 2 in the SI for details) and can well be fitted with a two-parameter model (Figure 4a) indicated by the first-order lag plot of the fitting (Figure 4b) showing a fully symmetrical “shotgun” pattern. The fitting returned two rate constants, 21668 ± 133 s^–1^ and 9291 ± 20 s^–1^. However, assignment of these two rate constants is not trivial without prior knowledge. By comparing these rates with those obtained by the FD method or the TD approach under direct excitation, we assigned these two rate values to *k*_31_ and 2*k*_5_^′^, respectively, yielding *k*_31_ = 21668 ± 133 s^–1^ and *k*_5_^′^ = 4645 ± 20 s^–1^. This means that this multiphotonic excitation TD approach, though providing a possibility in theory, fails in estimating the value of the radiative decay rate of state **2** (*k*_21_) of this sample with acceptable reliability and accuracy, probably due to a much smaller contribution of this parameter to the luminescence kinetics compared to the other two parameters. This originates from the difficulty in separating overlapping exponential decay functions in the fitting process.

**Figure 4:**
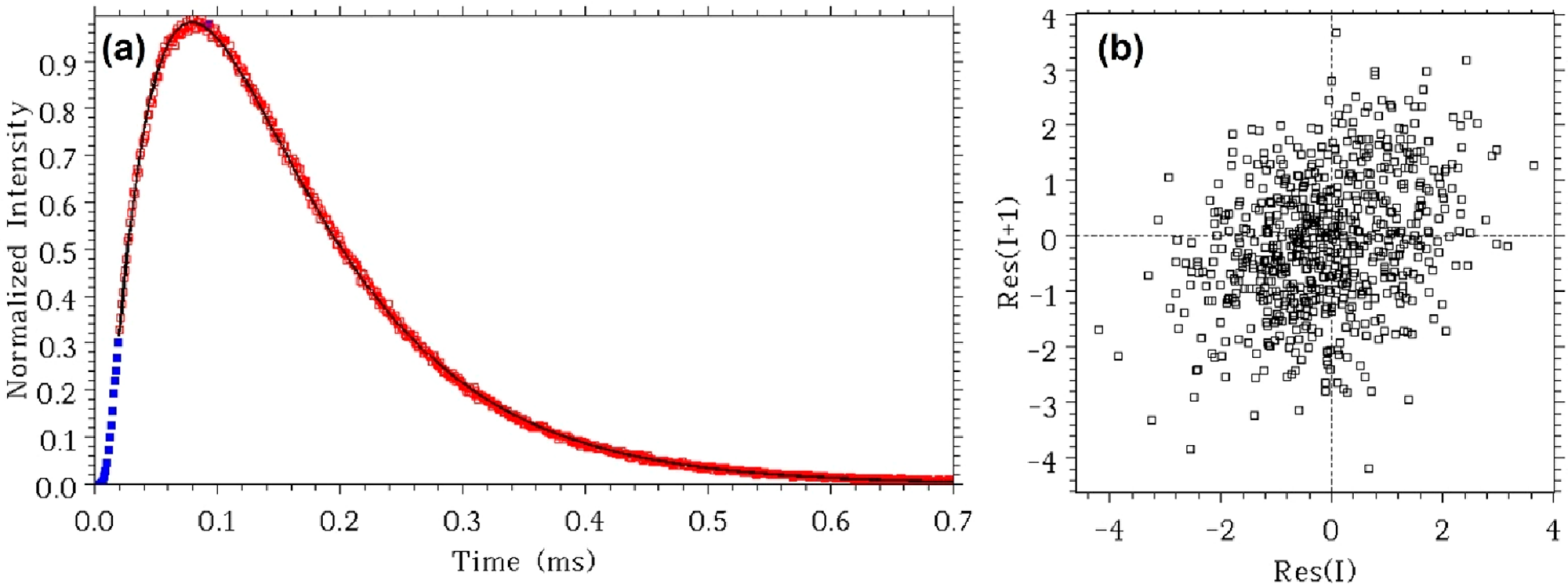
(a) Intensity decay in the time domain method of NaYF_4_:Yb,Er upconversion nanoparticles in OA at 543 nm under multiphotonic excitation at 978 nm (pulse width 20 μs). The black solid line is the fitted curve using two rate parameters. Red symbols are data points taken for fitting. (b) First-order lag plot of the fitting in (a) using equation S59′ using two rate parameters. The lag plot is a fully symmetrical “shotgun” pattern showing that two parameters are quite enough in the fitting process.

The obtained decay rates by the FD and TD methods are summarized in Table 1. It is worth emphasizing that the FD method can assess with sufficient reliability and accuracy the information about the decay rate of state **2** to return its combined decay rate *k*_21_^′^. However, the radiative decay rate *k*_21_ of state **2** may have been underestimated compared to previously reported values.^38−40^ According to eq 11, this does reveal that the escape process of state **2** under the current measurement conditions (inefficient quenching medium and high average excitation intensity) is strongly dominated by the ETU process rather than the linear decay process. This points at a limitation of the proposed FD method, i.e., the *k*_21_ could be underestimated if its contribution to the overall decay rate of state **2** is marginal compared to that of the upconversion term. The underestimation of *k*_21_ by the FD method is in line with the results obtained by the multiphotonic excitation TD approach, where *k*_21_ cannot be sought at all, indicating that *k*_21_ is indeed small for the current nanoparticles when dispersed in a less quenching solvent like oleic acid.^41^ It should be noted that the obtained *k*_31_ and *k*_5_^′^ from the FD measurements agree reasonably well with those obtained from the multiphotonic excitation TD measurements. Comparing the FD method with the direct excitation TD approach, the obtained *k*_5_^′^ values agree very well with each other, and the obtained *k*_31_ values also correlate reasonably well.

**Table 1:** Obtained Decay Rates of UCNPs Dispersed in Oleic Acid Obtained by the Frequency-Domain and Time-Domain Methods

Method	*k*_31_ (s^–1^)	*k*_5_^′^ (s^–1^)	*k*_21_^′^ (s^–1^)	*r*	*k*_21_ (s^–1^)
FD	18048 ± 174	6000 ± 30	450 ± 20	0.024	∼11
TD, direct excitation	∼14443	∼6140	-	-	-
TD, multiphotonic excitation	21668 ± 133	4645 ± 20	-	-	-

It should be pointed out that, under these excitation approaches, the extent to which the involved lanthanide excited states are populated is very different. In the FD approach, the excitation was moderate or strong due to the high average excitation intensity in use (110 W/cm^2^), while the direct and multiphotonic excitation TD approaches provided a relatively weak excitation condition on average due to the short pulse duration (10–20 μs). The difference in the obtained decay rates of the emitting and intermediate states again emphasize the collective and history-dependent feature of the luminescence kinetics of lanthanide-activated nanoparticles, essentially a result of the energy transfer networks.^20^ It suggests that, whenever discussing the luminescence kinetics of lanthanide luminescent nanoparticles, its excitation history needs to be taken into account.

It is known that the vibrational modes in the solvent molecules can quench lanthanide excited states in UCNPs,^41,42^ which will affect the UCL kinetics. We subsequently investigated the quenching effect of the solvent on UCNPs using the proposed FD method. FD measurements were performed on the same batch of UCNPs, but the UCNPs were now dispersed in H_2_O after removing the organic ligands on the surface. The quadrature data of the UCNPs in H_2_O, measured under an average excitation intensity of 110 W/cm^2^, can be well fitted with eq 16 (Figure S3a), yielding *k*_31_ = 34000 ± 630 s^–1^, *k*_5_^′^ = 8860 ± 90 s^–1^, and *k*_21_^′^ = 627 ± 20 s^–1^. Note that these values are remarkably larger than their respective values for the UCNPs dispersed in OA, which is expected due to the known, much stronger quenching effect of H_2_O to lanthanide excited states than for OA.^41,42^ It should also be noted that the fitting to the phase lag data (Figure S3d) returned a significantly larger *r* value (0.227) than in OA, yielding a *k*_21_ (= *rk*_21_^′^) value around 142 s^–1^, which is in good agreement with previously reported values for this parameter.^38−40^ Here, the result that *k*_21_ is better estimated from *r* and *k*_21_^′^ is also in line with the significantly larger contribution of the *k*_21_^′^ term to the quadrature data in this medium than in OA (see Figure S3a and Figure 2a).

In our previous work, the phase lag of UCNPs was proposed as an encoding dimension for optically multiplexing applications.^33^ Our current data show that the same UCNPs, but dispersed in different media, could exhibit very different phase lags (Figure 2d and Figure S3d). It suggests that the phase lag of UCNPs may be used as an identity for sensing applications after careful calibration.

The experimental results discussed above show that the results obtained using the FD method can correlate reasonably well with those obtained using the TD methods under either direct or multiphotonic excitation. The FD method can thus provide an alternative for characterizing the luminescence kinetics of UCNPs with good reliability and accuracy. It should be emphasized that the FD method can offer many advantages compared to the TD methods in characterizing UCNPs. For example, the FD method has lower requirements on instrumentation, not involving costly short-pulse lasers and single-photon counting detectors. Second, the signal-to-noise ratio of the luminescence signal can be high, because the material is continuously irradiated by the laser source with adequate power, so that weak emitting materials can also be well characterized. Third, the detection is based on the lock-in detection principle and is thus more insensitive to noise in the environment and from the instrument. In addition, the high stability of UCNPs that are unlikely to be photobleached even for longer and stronger excitation also makes them suitable for FD analysis. For TD methods, the direct excitation approach is experimentally demanding for UCNPs, because it requires specific excitation and detection wavelengths matching the respective absorption and emission bands. Moreover, the multiphotonic excitation approach suffers from low signal, due to the short excitation pulses that barely initiate the upconversion process, and thus it can often be difficult to characterize weak emitting nanoparticles. We also like to point out the fact that the two-photon upconversion rate-equation model presented in this work, the basis for both the FD and TD analyses, is rather simplified, overlooking and ignoring many details, e.g., solvent quenching effect. The limitation in the model may explain the fact that the fit to the TD data does not provide full concordance with the fit to the FD data. Adoption of a more sophisticated kinetic model^43^ can potentially improve the interpretation of the TD data but predictably will make the theoretical analysis on the FD response of UCNPs very challenging, or even impossible to reach an analytical expression, which is always insightful and thus valuable. Though with the limitation, the high-quality fitting to the FD data, as demonstrated in Figure 2 and Figure S3, does indicate that our model has adequately captured some key features of the studied upconversion luminescence process. In this sense, our simplified model may be interpreted as a valid equivalent model.

By theoretical analysis of the response of UCL to sinusoidal-wave excitation using rate equation models, we found that the frequency-domain (FD) method allows the effective decay rates of three critical energy levels involved in the most representative two-photon upconversion process to be obtained in a single experiment. This is supported by the good agreement between the results obtained by the FD and time-domain (TD) methods, under direct or multiphotonic excitation. The higher reliability of the fits utilizing the FD method is reflected by the smaller uncertainty of the determined decay rates in comparison to the data obtained by the TD methods. The reliability and accuracy of the FD method was experimentally demonstrated for the green upconversion emission of the Er^3+^ ions, and we assume it is also valid for other two-photon energy transfer upconversion processes, responsible for, e.g., the near-infrared emission (800 nm) of Tm^3+^ and the green emission of Ho^3+^ ions. Moreover, the lower requirements on instrumentation to apply the FD method will facilitate its further implementation. Our development of the FD method is expected to provide a powerful alternative to the TD method to characterize the nonlinear emission of upconversion materials, facilitating the exploitation of UCL kinetics and the underlying photophysics.

## Supporting Information Available

Detailed theoretical analysis on the sinusoidal-excitation response of two-photon upconversion emission, fitting of upconversion luminescence rise-decay profile, additional experimental details, and supplementary figures (PDF)
