# Supplementary material for: Frequency-Domain Method for Characterization of Upconversion Luminescence Kinetics
Source: J Phys Chem Lett. 2023 Apr 3;14(14):3436–44. doi: 10.1021/acs.jpclett.3c00269 (PMC10108355; doi:10.1021/acs.jpclett.3c00269)
Supplement: Supplementary file 1 — jz3c00269_si_001.pdf [file jz3c00269_si_001.pdf]

SUPPORTING INFORMATION

Frequency-Domain Method for Characterization of Upconversion Luminescence Kinetics

Lucía Labrador-Páez,[a] Jouko Kankare,[b] Iko Hyppänen,[b] Tero Soukka,[c,*] Elina Andresen,[d] Ute Resch-Genger,[d] Jerker Widengren,[a] Haichun Liu[a,*]

[a] *Department of Applied Physics, KTH Royal Institute of Technology, SE-10691 Stockholm, Sweden.*

[b] *Department of Chemistry, University of Turku, FI-20014 Turku, Finland.*

[c] *Department of Life Technologies/Biotechnology, University of Turku, FI-20520 Turku, Finland.*

[d] *Division of Biophotonics, Federal Institute for Materials Research and Testing (BAM), Richard-Willstätter-Str. 11, 12489 Berlin, Germany.*

**haichun@kth.se; tejoso@utu.fi.*

Contents:

Section 1. Theoretical analysis on the sinusoidal-excitation response of the standard two-photon upconversion emission

Section 2. Fitting of upconversion luminescence rise-decay profile under multi-photonic excitation

Section 3. Experimental methods

Section 1. Theoretical analysis on the sinusoidal-excitation response of the standard two-photon upconversion emission

Jouko Kankare

According to the upconversion kinetics model developed in a previous work,[1] the system schematized in **Fig. 1b** in the main text can be described by a set of differential equations:

$$\frac{dn_5}{dt} = k_{45}n_4 - W_{51}n_1n_5 - W_{52}n_2n_5 - k_{54}n_5 \qquad (S1')$$

$$\frac{dn_2}{dt} = W_{51}n_1n_5 - W_{52}n_2n_5 - k_{21}n_2 \qquad (S2')$$

$$\frac{dn_3}{dt} = W_{52}n_2n_5 - k_{31}n_3 \qquad (S3')$$

Here n_i is the population density of state i, k_{ij} are the decay rate from state i to state j, and W_{ij} are the energy transfer rate from state i to state j. It is assumed that the population densities of the ground states, 1 and 4, are constants. The pumping rate k_{45} is given by:

$$k_{45} = P\sigma F(t) \qquad (S4')$$

where P is the excitation photon flux, σ is the absorption cross-section of the sensitizer, and $F(t)$ is the normalized dimensionless modulation function of excitation light.

In the following, the constant terms are combined:

$$k'_{45} = P\sigma n_4 \qquad (S5')$$

$$k'_5 = k_{54} + W_{51}n_1 \qquad (S6')$$

$$W'_{51} = W_{51}n_1 \qquad (S7')$$

At this moment we assume that the term $W_{52}n_2$ in Eq. (S1') is small compared to k'_5 and we ignore it.

That depends on the ratio

$$\frac{W_{52}n_2}{k'_5} = \frac{W_{52}n_2}{k_{54} + W_{51}n_1} \ll 1 \qquad (S8')$$

This is generally valid, because of $n_2 \ll n_1$ when the excitation intensity is not too high. Further support for this assumption is given later, in Eq. (S53'). The approximation results in the rate-equation model as

$$\frac{dn_5}{dt} = k'_{45}F(t) - k'_5n_5 \qquad (S9')$$

$$\frac{dn_2}{dt} = W'_{51}n_5 - W_{52}n_2n_5 - k_{21}n_2 \qquad (S10')$$

$$\frac{dn_3}{dt} = W_{52}n_2n_5 - k_{31}n_3 \qquad (S3')$$

The standard solution of the first order linear differential equation can be used to get the solutions of this system. When a differential equation is written in the form

$$\frac{dy}{dt} + P(t)y = Q(t) \qquad (S11')$$

its solution is given by

$$y(t) = \exp\left(-\int_0^t P(t)dt\right)\left[y(0) + \int_0^t \exp\left(\int_0^t P(t')dt'\right)Q(t)dt\right] \qquad (S12')$$

Note that in the present upconversion system $P(t)$ represents the overall decay rate of the state of interest while $Q(t)$ stands for the pumping rate. This is now applied to the simplified Eq. (S9') giving

$$n_5(t) = \exp(-k_5' t)\left[n_5(0) + k_{45}' \int_0^t \exp(k_5' t)F(t)dt\right] \qquad (S13')$$

Into Eq. (S13') substituting the modulation function

$$F(t) = 1 + \mu \cos \omega t \qquad (S14')$$

with μ as the modulation degree ($0 < \mu \leq 1$) and defining $\alpha_5 = \frac{\omega}{k_5'}$ and $\varphi_5 = \arctan \alpha_5$ gives

$$\begin{aligned} n_5(t) &= \exp(-k_5' t)\left[n_5(0) + k_{45}' \int_0^t \exp(k_5' t')(1 + \mu \cos \omega t')dt'\right] \\ &= n_5(0)\exp(-k_5' t) + \frac{k_{45}'}{k_5'}(1 - \exp(-k_5' t)) - \frac{\mu k_{45}' k_5'}{k_5'^2 + \omega^2} e^{-k_5' t} \\ &\quad + \frac{\mu k_{45}'}{k_5'^2 + \omega^2}[k_5' \cos \omega t + \omega \sin \omega t] \\ &= \frac{k_{45}'}{k_5'} - \left[\frac{k_{45}'}{k_5'} + \frac{\mu k_{45}' k_5'}{k_5'^2 + \omega^2} - n_5(0)\right]\exp(-k_5' t) + \frac{\mu k_{45}' \cos(\omega t - \varphi_5)}{\sqrt{k_5'^2 + \omega^2}} \end{aligned} \qquad (S15')$$

In the FD method the measurements are performed far from the initial point ($t = 0$) and consequently the exponentially decaying terms are ignored. In addition, by defining

$$A = \frac{k_{45}'}{k_5'} \qquad (S16')$$

Eq. (S15') becomes

$$n_5(t) = A\left[1 + \frac{\mu \cos(\omega t - \varphi_5)}{\sqrt{1 + \alpha_5^2}}\right] \qquad (S17')$$

The physical meaning of Eq. (S17') is that that under the modulated excitation specified in Eq. (S14'), the population of the sensitizer excited state would exhibit a sinusoidal oscillation with the same frequency but with a phase lag φ_5 and a demodulation factor $1/\sqrt{1 + \alpha_5^2}$.

Approximation II (weak excitation)

The solution of the equations (S10') and (S3') is now divided into two parts according to the level of approximation. In the weak excitation approximation, **App II**, it is assumed that the nonlinear term $W_{52}n_2n_5$ in Eq. (S10') is neglected. This means

$$\frac{W_{52}n_5}{k_{21}} \approx \frac{W_{52}A}{k_{21}} = \frac{W_{52}k_{45}'}{k_{21}k_5'} \ll 1 \qquad (S18')$$

This approximation is obviously valid if the excitation intensity is low enough, in conformity with Eq. (S8’). In **App II** Eq. (S10’) is solved by using formula (S12’). Then $P(t) = k_{21}$ and $Q(t) = W'_{51}n_5$ and

$$n_2(t) = e^{-k_{21}t}\left[n_2(0) + AW'_{51}\int_0^t e^{k_{21}t}\left(1 + \frac{\mu\cos(\omega t - \varphi_5)}{\sqrt{1+\alpha_5^2}}\right)dt\right]$$

$$\approx \frac{AW'_{51}}{k_{21}} + \mu AW'_{51}\frac{k_{21}\cos(\omega t - \varphi_5) + \omega\sin(\omega t - \varphi_5)}{\sqrt{1+\alpha_5^2}}$$

$$= \frac{AW'_{51}}{k_{21}}\left[1 + \frac{\mu\cos(\omega t - \varphi_5 - \varphi_{21})}{\sqrt{(1+\alpha_5^2)(1+\alpha_{21}^2)}}\right] \qquad (S19')$$

For the solution of **App II**, see equations after (S30’).

Approximation I (medium excitation)

In this approximation the condition (S18’) is not valid, i.e. the excitation is stronger but still keeping it low enough for the condition (S8’) to be valid. Eq. (S10’) can then be solved by using formula (S12’) with $P(t) = k_{21} + W_{52}n_5$ and $Q(t) = W'_{51}n_5$. Now by applying Eqs. (S11’) and (S12’) and defining $k'_{21} = k_{21} + AW_{52}$, it yields

$$\int_0^t Pdt = \int_0^t (k_{21} + W_{52}n_5)dt = k'_{21}t + \frac{\mu AW_{52}}{\sqrt{1+\alpha_5^2}}\int_0^t \cos(\omega t - \varphi_5)\,dt$$

$$= k'_{21}t + \frac{\mu AW_{52}}{\omega\sqrt{1+\alpha_5^2}}[\sin\varphi_5 + \sin(\omega t - \varphi_5)]$$

$$= k'_{21}t + \frac{\mu AW_{52}}{k'_5(1+\alpha_5^2)} + \gamma\sin\theta(t) \qquad (S20')$$

Here it was defined that $\theta(t) = \omega t - \varphi_5$ and $\gamma = \frac{\mu AW_{52}}{\omega\sqrt{1+\alpha_5^2}} = \frac{\mu k'_{45}W_{52}}{\omega\sqrt{k_5'^2+\omega^2}}$. According to Eq. (S12’):

$$\exp\left(\int_0^t Pdt\right) = e^{k'_{21}t}\exp\left(\frac{\mu AW_{52}}{k'_5(1+\alpha_5^2)}\right)e^{\gamma\sin\theta(t)} \qquad (S21')$$

The following truncated series expansion is utilized

$$e^{\gamma\sin\theta} \approx 1 + \gamma\sin\theta \qquad (S22')$$

where the first order approximation in terms of γ is taken, assuming that $\gamma \ll 1$. As seen in the definition of γ, this assumption means that the excitation intensity is not too high and ω is not too low.

In order to examine the validity of this assumption, the value of γ is evaluated using typical values for the involved parameters as listed in Table S1.

Table S1 The parameter values used to evaluate γ

μ	I_{exc} (W cm^{-2})	σ (cm^2)	n_4 (cm^{-3})	n_1 (cm^{-3})	k_{54} (s^{-1})	W_{51} (cm^3 s^{-1})	W_{52} (cm^3 s^{-1})
0.1	110	1.7x10^{-20}	1.3x10^{21}	2.3x10^{20}	1000	1x10^{-17}	1x10^{-17}

The dependence of γ on frequency ($\omega/2\pi$) was numerically calculated and is presented in Fig. S1. As seen, the γ value is rather small (<0.023) when the frequency is higher than 50 Hz, supporting our assumption.

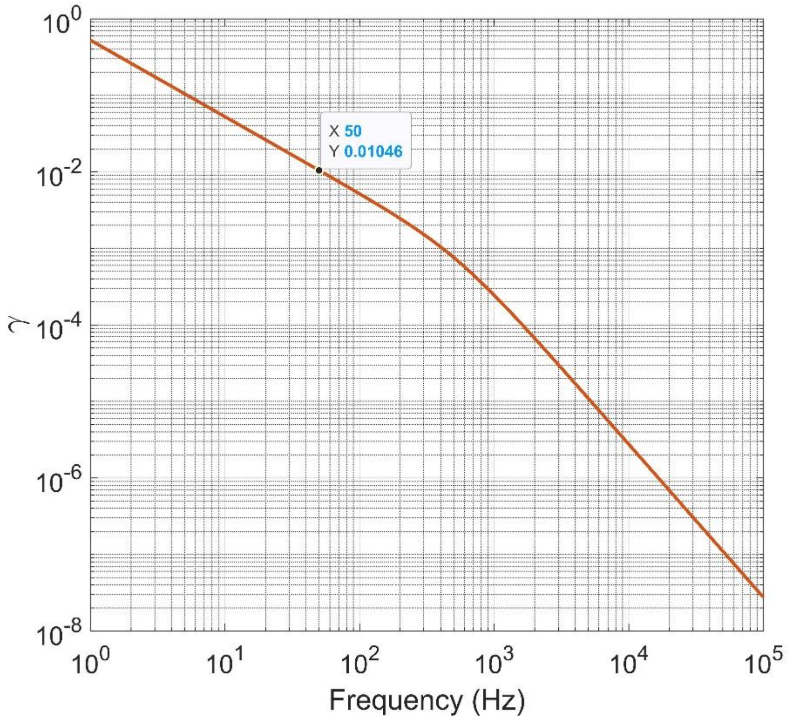

Figure S1 The dependence of γ on frequency ($\omega/2\pi$)

From Eq. (S12') it is obtained (assuming y(0)=0) that

$$n_2(t) = \exp\left(-\int_0^t Pdt\right)\int_0^t \exp\left(\int_0^t Pdt'\right) Q(t)dt$$

$$= AW'_{51}e^{-\gamma\sin\theta}e^{-k'_{21}t}\int_0^t e^{k'_{21}t}\,e^{\gamma\sin\theta(t)}\left\{1+\frac{\mu\cos\theta(t)}{\sqrt{1+\alpha_5^2}}\right\}dt \quad (S23')$$

Here the constant term $\exp\left(\frac{\mu AW_{52}}{k'_5(1+\alpha_5^2)}\right)$ was eliminated. The first integral in evaluating Eq. (S23') is

$$AW'_{51}e^{-\gamma\sin\theta}e^{-k'_{21}t}\int_0^t e^{k'_{21}t}\,e^{\gamma\sin\theta(t)}dt \cong AW'_{51}(1-\gamma\sin\theta)e^{-k'_{21}t}\int_0^t e^{k'_{21}t}\,(1+\gamma\sin\theta(t))dt$$

$$\xrightarrow{t\to\infty}\frac{AW'_{51}}{k'_{21}}(1-\gamma\sin\theta)\left[1+\frac{\gamma k'_{21}}{k'^2_{21}+\omega^2}(k'_{21}\sin\theta-\omega\cos\theta)\right]$$

$$\xrightarrow{\gamma^2\cong 0}\frac{AW'_{51}}{k'_{21}}\left[1+\gamma\left(\frac{k'^2_{21}}{k'^2_{21}+\omega^2}-1\right)\sin\theta-\frac{\gamma k'_{21}\omega}{k'^2_{21}+\omega^2}\cos\theta\right]$$

$$=\frac{AW'_{51}}{k'_{21}}\left[1-\frac{\gamma\omega}{k'^2_{21}+\omega^2}(\omega\sin\theta+k'_{21}\cos\theta)\right] \qquad (S24')$$

and the second integral in Eq. (S23')

$$AW'_{51}e^{-k'_{21}t}e^{-\gamma\sin\theta}\int_0^t e^{k'_{21}t}\,e^{\gamma\sin\theta(t)}\frac{\mu\cos\theta(t)}{\sqrt{1+\alpha_5^2}}dt$$

$$=\frac{\mu AW'_{51}e^{-k'_{21}t}e^{-\gamma\sin\theta}}{\sqrt{1+\alpha_5^2}}\int_0^t e^{k'_{21}t}\,e^{\gamma\sin\theta(t)}\cos\theta(t)dt \qquad (S25')$$

Because of

$$\frac{d}{dt}e^{\gamma\sin\theta(t)}=\omega\gamma e^{\gamma\sin\theta(t)}\cos\theta(t) \qquad (S26')$$

and $\omega\gamma=\mu AW_{52}/\sqrt{1+\alpha_5^2}$, it can be obtained that

$$\frac{\mu AW'_{51}e^{-k'_{21}t}e^{-\gamma\sin\theta}}{\sqrt{1+\alpha_5^2}}\int_0^t e^{k'_{21}t}\,e^{\gamma\sin\theta(t)}\cos\theta(t)dt$$

$$=\frac{W'_{51}}{W_{52}}e^{-k'_{21}t}e^{-\gamma\sin\theta}\int_0^t e^{k'_{21}t}\frac{d}{dt}e^{\gamma\sin\theta(t)}dt \qquad (S27')$$

By using integration by parts, it is obtained that

$$\int_0^t e^{k'_{21}t}\frac{d}{dt}e^{\gamma\sin\theta(t)}dt=\left[e^{k'_{21}t}e^{\gamma\sin\theta(t)}\right]_0^t-k'_{21}\int_0^t e^{k'_{21}t}e^{\gamma\sin\theta(t)}dt$$

$$=e^{k'_{21}t}e^{\gamma\sin\theta(t)}-e^{-\gamma\sin\varphi_5}-k'_{21}\int_0^t e^{k'_{21}t}e^{\gamma\sin\theta(t)}dt \qquad (S28')$$

Substituting Eq. (S28') into Eq. (S27') gives

$$\frac{W'_{51}}{W_{52}}e^{-k'_{21}t}e^{-\gamma\sin\theta}\int_0^t e^{k'_{21}t}\frac{d}{dt}e^{\gamma\sin\theta}dt$$

$$=\frac{W'_{51}}{W_{52}}\left\{1-e^{-k'_{21}t}e^{-\gamma(\sin\theta+\sin\varphi_5)}-k'_{21}e^{-k'_{21}t}e^{-\gamma\sin\theta}\int_0^t e^{k'_{21}t}e^{\gamma\sin\theta(t)}dt\right\}$$

$$\xrightarrow{t\to\infty} \frac{\mu A W'_{51}}{(k'^2_{21}+\omega^2)\sqrt{1+\alpha_5^2}}(\omega\sin\theta + k'_{21}\cos\theta) \qquad (S29')$$

Substitution of Eq. (S29') and Eq. (S24') into Eq. (S23') yields

$$n_2(t) = \frac{AW'_{51}}{k'_{21}}\left[1-\frac{\omega\gamma}{k'^2_{21}+\omega^2}(\omega\sin\theta + k'_{21}\cos\theta)\right] + \frac{\mu A W'_{51}}{(k'^2_{21}+\omega^2)\sqrt{1+\alpha_5^2}}(\omega\sin\theta + k'_{21}\cos\theta)$$

$$= \frac{AW'_{51}}{k'_{21}}\left\{1-\frac{\omega\gamma}{k'^2_{21}+\omega^2}(\omega\sin\theta + k'_{21}\cos\theta)\right.$$

$$\left. + \frac{\mu k'_{21}}{(k'^2_{21}+\omega^2)\sqrt{1+\alpha_5^2}}(\omega\sin\theta + k'_{21}\cos\theta)\right\}$$

$$= \frac{AW'_{51}}{k'_{21}}\left[1+\frac{\mu(\omega\sin\theta + k'_{21}\cos\theta)}{(k'^2_{21}+\omega^2)\sqrt{1+\alpha_5^2}}(k'_{21}-AW_{52})\right]$$

$$= \frac{AW'_{51}}{k'_{21}}\left[1+\frac{\mu k_{21}}{(k'^2_{21}+\omega^2)\sqrt{1+\alpha_5^2}}(\omega\sin\theta + k'_{21}\cos\theta)\right] \qquad (S30')$$

By defining $\varphi_{21} = \arctan\frac{\omega}{k'_{21}}$ and $r = k_{21}/k'_{21}$, it can be obtained that

$$n_2(t) = \frac{AW'_{51}}{k'_{21}}\left(1+\frac{\mu r}{\sqrt{(1+\alpha_5^2)(1+\alpha_{21}^2)}}\cos(\omega t-\varphi_5-\varphi_{21})\right) \qquad (S31')$$

The physical meaning of Eq. (S31') is that the population of the intermediate state of the activator (state 2) would exhibit an extra phase lag φ_{21} and an extra demodulation factor $r/\sqrt{(1+\alpha_{21}^2)}$ associated with the decay property of this state, compared to those of state 5. This makes sense considering that state 2 is populated via state 5 by energy transfer. Note Eq. (S31') becomes exactly the same as Eq. (S19') in **App II** when $r\to 1$ and $k'_{21}\to k_{21}$. With the corresponding replacements the subsequent equations in this paper are valid.

Next, we solve Eq. (S3') to determine n_3, representing the harmonic upconversion emission signal, based on the following considerations. In FD experiments the harmonic emission signal is recorded on a dual-phase lock-in amplifier, with both the in-phase signal S_x and out-of-phase (quadrature) signal S_y recorded. It is mathematically convenient to treat the entire signal as a complex quantity:

$$S(\omega) = S_x(\omega) - iS_y(\omega) \qquad (S32')$$

Ideally, ignoring the instrumental time constant, the in-phase and out-of-phase signals can be considered as time averages over infinite time. In the present case, including the instrument gain factor g, the in-phase and quadrature signals become

$$S_x = g \lim_{T\to\infty} \frac{1}{T} \int_0^T f(t) \cos \omega t \, dt \qquad (S33')$$

$$S_y = g \lim_{T\to\infty} \frac{1}{T} \int_0^T f(t) \sin \omega t \, dt \qquad (S34')$$

where $f(t)$ denotes the time-dependent signal.

Taking into account Eq. (S32'):

$$S(\omega) = S_x(\omega) - iS_y(\omega) = g \lim_{T\to\infty} \frac{1}{T} \int_0^T e^{-i\omega t} f(t) dt = g\boldsymbol{L}\{f(t)\} \qquad (S35')$$

Here is defined a lock-in transform $\boldsymbol{L}\{\}$ as a short-hand notation. Function $f(t)$ should be a periodic function with the same frequency ω, otherwise the application of $\boldsymbol{L}\{\}$ to $f(t)$ results in zero. We are now looking for the signal

$$S(\omega) = g\boldsymbol{L}\{n_3\} \qquad (S36')$$

Transform $\boldsymbol{L}\{\}$ is now applied to both sides of Eq. (S3'):

$$\boldsymbol{L}\left\{\frac{dn_3}{dt}\right\} = W_{52}\boldsymbol{L}\{n_2 n_5\} - k_{31}\boldsymbol{L}\{n_3\} \qquad (S37')$$

Integration by parts gives

$$\boldsymbol{L}\left\{\frac{dn_3}{dt}\right\} = \lim_{T\to\infty} \frac{1}{T} \int_0^T e^{-i\omega t} \frac{dn_3}{dt} dt = \lim_{T\to\infty} \frac{i\omega}{T} \int_0^T e^{-i\,\omega t} n_3 dt = i\omega \boldsymbol{L}\{n_3\} \qquad (S38')$$

Substitution into Eq. (37') gives

$$\boldsymbol{L}\{n_3\} = \frac{W_{52}}{k_{31} + i\omega} \boldsymbol{L}\{n_2 n_5\} \qquad (S39')$$

From Eqs (S17') and (S31'):

$$n_2 n_5 = \frac{k_{45}'^2 W_{51}'}{k_5'^2 k_{21}} \left[1 + \frac{\mu r}{\sqrt{(1+\alpha_{21}^2)(1+\alpha_5^2)}} \cos(\omega t - \varphi_2 - \varphi_5) + \frac{\mu \cos(\omega t - \varphi_5)}{\sqrt{1+\alpha_5^2}} \right.$$
$$\left. + \frac{\mu^2 r}{(1+\alpha_5^2)\sqrt{(1+\alpha_{21}^2)}} \cos(\omega t - \varphi_2 - \varphi_5) \cos(\omega t - \varphi_5) \right] \qquad (S40')$$

Using Eq. (S36') the application of the lock-in transform gives

$$\boldsymbol{L}\{n_2 n_5\} = \frac{\mu k_{45}'^2 W_{51}'}{2k_5'^2 k_{21}}\left[\frac{r}{(1+i\alpha_{21})(1+i\alpha_5)} + \frac{1}{1+i\alpha_5}\right] \tag{S41'}$$

The output signal can now be solved from Eq. (S39'):

$$S(\omega) = g\frac{\mu k_{45}'^2 W_{51}' W_{52}}{2k_5'^2 k_{21} k_{31}}\left[\frac{1}{(1+i\alpha_{31})(1+i\alpha_5)}\left(1+\frac{r}{1+i\alpha_{21}}\right)\right] \tag{S42'}$$

This expression can be written in different ways. In many applications it is feasible to use the signal in a polar form, *i.e.* in terms of modulus and phase. We have

$$\frac{1}{1+i\alpha_5} = \frac{e^{-i\varphi_5}}{\sqrt{1+\alpha_5^2}}\ ;\ \frac{1}{1+i\alpha_{31}} = \frac{e^{-i\varphi_{31}}}{\sqrt{1+\alpha_{31}^2}} \tag{S43'}$$

The third factor in Eq. (S42') becomes

$$1+\frac{r}{1+i\alpha_{21}} = 1+r\frac{1-i\alpha_{21}}{1+\alpha_{21}^2} = \sqrt{\frac{(1+r)^2+\alpha_{21}^2}{1+\alpha_{21}^2}}\,e^{-i\varphi_{21}} \tag{S44'}$$

where

$$\tan\varphi_{21} = \frac{r\alpha_{21}}{1+r+\alpha_{21}^2} \tag{S45'}$$

Hence the signal in the polar mode is

$$S(\omega) = g\frac{\mu k_{45}'^2 W_{51}' W_{52}}{2k_{31} k_5'^2 k_{21}'}\sqrt{\frac{(1+r)^2+\alpha_{21}^2}{(1+\alpha_{31}^2)(1+\alpha_5^2)(1+\alpha_{21}^2)}}\,e^{-i\Phi} \tag{S46'}$$

Here Φ is the total phase lag of the emission signal:

$$\Phi = \varphi_{31}+\varphi_5+\varphi_{21} = \arctan\alpha_{31} + \arctan\alpha_5 + \arctan\frac{r\alpha_{21}}{1+r+\alpha_{21}^2} \tag{S47'}$$

Eq. (S42') can be written as

$$S(\omega) = g\frac{\mu k_{45}'^2 W_{51}' W_{52}}{2k_5' k_{21}}\left(\frac{h_{31}}{k_{31}+i\omega} + \frac{h_5}{k_5'+i\omega} + \frac{h_{21}}{k_{21}'+i\omega}\right) \tag{S48'}$$

where

$$h_{31} = \frac{rk_{21}'}{(k_{31}-k_{21}')(k_{31}-k_5')} - \frac{1}{k_{31}-k_5'}$$

$$h_5 = \frac{rk_{21}'}{(k_{21}'-k_5')(k_{31}-k_5')} + \frac{1}{k_{31}-k_5'}$$

$$h_{21} = \frac{rk'_{21}}{(k_{31} - k'_{21})(k'_5 - k'_{21})} \qquad (S49')$$

and

$$h_{31} + h_5 + h_{21} = 0 \qquad (S50')$$

From Eq. (S48’) we obtain the quadrature component

$$S_y(\omega) = g\frac{\mu k'^2_{45} W'_{51} W_{52}}{2k'_5 k'_{21}}\left(\frac{\omega h_{31}}{k^2_{31} + \omega^2} + \frac{\omega h_5}{k'^2_5 + \omega^2} + \frac{\omega h_{21}}{k'^2_{21} + \omega^2}\right) \qquad (S51')$$

and the in-phase component

$$S_x(\omega) = g\frac{\mu k'^2_{45} W'_{51} W_{52}}{2k'_5 k'_{21}}\left(\frac{k_{31} h_{31}}{k^2_{31} + \omega^2} + \frac{k'_5 h_5}{k'^2_5 + \omega^2} + \frac{k'_{21} h_{21}}{k'^2_{21} + \omega^2}\right) \qquad (S52')$$

In order to check the internal consistency of **App I**, the condition (S8’) is evaluated by using Eq. (S31’):

$$\frac{W_{52} n_2}{k_{54} + W_{51} n_1} \approx \frac{W_{52} A W_{51} n_1}{(k_{54} + W_{51} n_1) k'_{21}} = \frac{(1 - r) W_{51} n_1}{k_{54} + W_{51} n_1} < 1 \qquad (S53')$$

Hence the condition (S8’) is valid at least to some extent depending on the numerical values of variables in (S53’).

Fitting procedure in the frequency-domain method

On recording the emission signal, the magnitude and phase lag on the one hand and in-phase and quadrature signals on the other hand are alternative and, in principle, equivalent methods. This is true in the absence of noise, but due to different weighing of data, noise can cause significant differences. The noise in the primary output signals of a lock-in amplifier, i.e., the in-phase and quadrature signals, is relatively constant or at least predictable within the entire frequency range of the instrument. The phase is secondary signal, derived from the ratio of in-phase and quadrature signals, and generally much more sensitive to noise. If these signals are small, their ratio becomes strongly erratic due to the noise. The external synchronous noise, such as that caused by scattering or prompt fluorescence in the measured sample, typically has a strong influence on the phase and on the in-phase signal, but less on the quadrature data. Consequently, the method of choice is typically recording the quadrature signal and do model fitting on the basis of these data. However, if the Kramers-Kronig transform of the quadrature signal fits well with the in-phase signal, indicating less external interference, then the additional use of in-phase signal is justified if considered useful on the statistical basis.

If the original set of differential equations is linear as, for instance, in the case of luminescent lanthanide complexes, the emission signal when recorded on a dual-phase lock-in amplifier, has the form of equation (S48’), generally[8,10]

$$S(\omega) = K\sum_{j=1}^{N}\frac{h_j}{k_j + i\omega} = K\sum_{j=1}^{N}\frac{h_j k_j}{k_j^2 + \omega^2} - iK\sum_{j=1}^{N}\frac{h_j \omega}{k_j^2 + \omega^2} \qquad (S54')$$

Here, all the constant coefficients are collected in K. Also the h-coefficients do not depend on frequency but instead they fulfill the condition

$$\sum_{j=1}^{N} h_j = 0 \qquad (S55')$$

The partial linearity of equation (S54’) suggests to use the fitting method whereby the rate constants k_j are varied according to some strategy and the coefficients Kh_j are determined by general linear least squares fitting. There are several ways how this fitting of experimental emission data to equation (S54’) could be done. We might use only the quadrature signal fitted to the imaginary part of equation (S54’) or the in-phase signal to the real part or simultaneously the in-phase and quadrature signals to the both parts of equation (S54’). In addition, we have to decide whether we allow the h-coefficients to vary freely or whether to apply the Bayesian condition (S55’) as a constraint in the fitting process. Mathematically the results should be the same but the presence of experimental noise changes the situation as stated above. The advantage of keeping h’s free of constraint (S55’) during the fitting process is that the sum of the resulting h-coefficients may indicate the validity of the assumed mechanism. On the other hand, by applying the constraint (S55’) the number of degrees of freedom decreases with concomitant benefits.

The prefactor K is generally not only a combination of rate coefficients but also of unknown instrumental gain and scaling factors, the intensity of excitation and the concentration of luminescent species. In order to compare visually the signal versus frequency curves measured at different conditions, it is worthwhile to cancel the influence of K by normalization. The obvious normalization factor is the total emission intensity:

$$S(0) = K\sum_{j=1}^{N} \frac{h_j}{k_j} \qquad (S56')$$

The use of this equation implies the determination of Kh_j and k_j but actually the numerical value for $S(0)$ can be estimated also directly from the experimental data without any assumption on the validity of equation (S54’)[10]. As a matter of fact, this is exactly the same way to normalize as done by Lakowicz.[3]

The set of equations (S1’) to (S3’) with its several nonlinear components does not give any clue that the solution could be expanded into the form of equation (S54’). However, it turned out that by using only three rate coefficients equation (S54’) represents fairly well the experimental observations. A perfect fit cannot be expected due to the simplistic character of the model and the required approximations. It turned out that the closed-form solution for the set (S1’), (S2’) and (S3’) could not be derived without approximations. The required presumption is that the excitation intensity is weak, meaning that the changes in the population densities n_1 and n_4 are negligible and they can be kept constant.

The fitting of experimentally recorded in-phase or quadrature data to equation (S54’) with the constraint (S55’) on the h-coefficients, will yield the three critical decay rates of the upconversion process, i.e., k_{31}, k_5', and k_{21}'. It should be noted that k_5' represents an effective decay rate of state 5 that groups all the depopulation processes affecting state 5 according to equation (S6’). Note parameter r is the ratio k_{21}/k_{21}', where k_{21} is the radiative decay rate through the transition of $^4I_{13/2}\rightarrow{}^4I_{15/2}$ and k_{21}' is the observed combined rate constant $k_{21}' = k_{21} + \frac{k_{45}' W_{52}}{k_5'}$. There are several ways how r and k_{21} can be extracted from the experimental results. The most straightforward method is to plot k_{21}' versus excitation photon flux P (proportional to excitation intensity), as the factor k_{45}' is directly proportional to P. Unfortunately, due to the lack of a calibrated light source, this method could not be used. The second method is to use the h-coefficients (S45’) and consider:

$$\left(\frac{Kh_5}{Kh_{21}}\right)_{\text{obs}} = s = \frac{h_5}{h_{21}} = \frac{(k_{31} - k_{21}')(k_5' - k_{21}' - rk_{21}')}{k_{21}' r (k_{31} - k_5')} \qquad (S57')$$

This can be solved for *r*:

$$r = \frac{(k_{31} - k'_{21})(k'_5 - k'_{21})}{k'_{21}[k_{31} - k'_{21} + s(k_{31} - k'_5)]} \qquad (S58')$$

The third method is based on the experimental overall phase lag, Φ, of UCL and equation (S52'). In this equation *r* is varied until the sum of squares of the differences between the experimental Φ and the right part of (S52') is minimized in the measured frequency range.

Section 2. Fitting of upconversion luminescence rise-decay profile under multi-photonic excitation

Data fitting of the upconversion luminescence rise-decay profile under multi-photonic excitation was done by minimizing the merit function

$$\chi^2 = \sum_i^N \frac{1}{\sigma_i^2}\left[y_i - \sum_j^M h_j e^{-k_j t_i}\right]^2 \tag{S59'}$$

Data points y_i are photon counts following approximately the Poisson distribution at least at the low intensity. In case of Poisson distribution variance σ_i^2 equals to mean and even if the binomial distribution is more valid, mean and variance are directly proportional.[4] At a higher intensity the distribution converges to Gaussian with the Poisson variance.[2] In the present case the estimate of mean was obtained by applying the Savitzky-Golay filter to data using an 11-point window.[5] The rate constants k_j were varied by using Chandler's direct search method STEPIT[6] and finalizing the minimum by linear regression. The errors were estimated by using the Monte Carlo bootstrap method.[2]

One of the most important problems in fitting exponential sums to data is the value of M, i.e. how many exponentials should be taken. Obviously, if the model to be simulated presents a constraint for M, there is no problem. One way to tackle the problem is to study whether the differences between the observed and calculated data points are randomly distributed and show very little autocorrelation. A simple tool for this purpose is the lag plot of residuals[7] which in the random case should show up as a "shotgun" pattern, a more or less symmetric distribution of points around the origin. A fully symmetric "shotgun" hit means that it is highly improbable to improve the fit by increasing the number of parameters. In the present case the residuals were "normalized" by dividing by standard deviation σ_i.

Section 3. Experimental methods

Frequency-domain (FD) measurements. The setup employed for FD measurements is described in detail in references [8, 9]. In brief, the excitation laser diode (RLT981000G, Roithner Lasertechnik) was sinusoidally modulated (LDTC2/2 Laser diode & Temperature controller, Wavelength Electronics). The emission band of interest was filtered (HQ520/20 or FL543/10, Chroma Technology and Thorlabs, respectively) and its intensity was detected by a photomultiplier tube (R1465, Hamamatsu). The signal was analyzed by a lock-in amplifier (SR830, Stanford Research Systems). The fitting of the data obtained by the FD method to the developed model was performed following the method described in reference [8].

Time-domain (TD) measurements. TD measurements were carried out on a spectrofluorometer (Edinburgh Instruments, FLS980-xD2-stm) equipped with an electrically pulsed 978 nm (8 W, long square pulses, pulse width of 20 µs) or 485 nm (100 mW, pulse width 10 µs) laser diode. The decay kinetics of the 543 nm emissions of Er^{3+} ions and the 1010 nm emission of Yb^{3+} ions under multi-photonic or direct excitation were recorded with a red-sensitive photomultiplier tube (H10720-20, Hamamatsu), connected to a time-correlated single photon counting (TCSPC) unit.

Supplementary figures

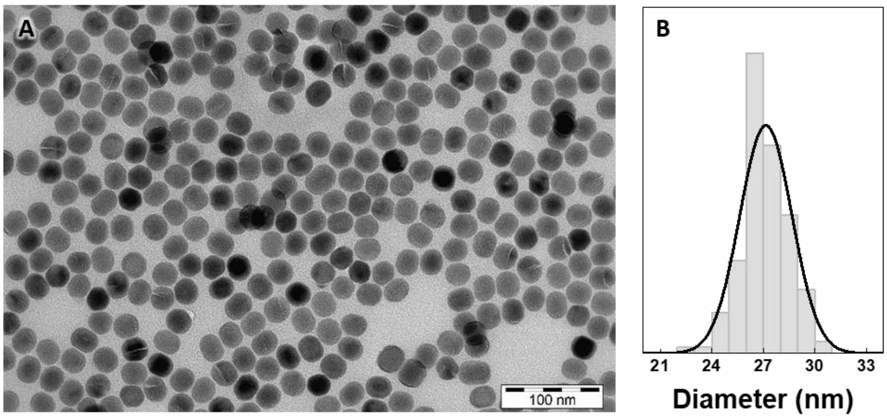

Figure S2 (A) Transmission electron microscopy image of $NaYF_4$:17%Yb, 3%Er UCNPs. **(B)** Size histogram from analysis on the nanoparticles in the image in **(A)**.

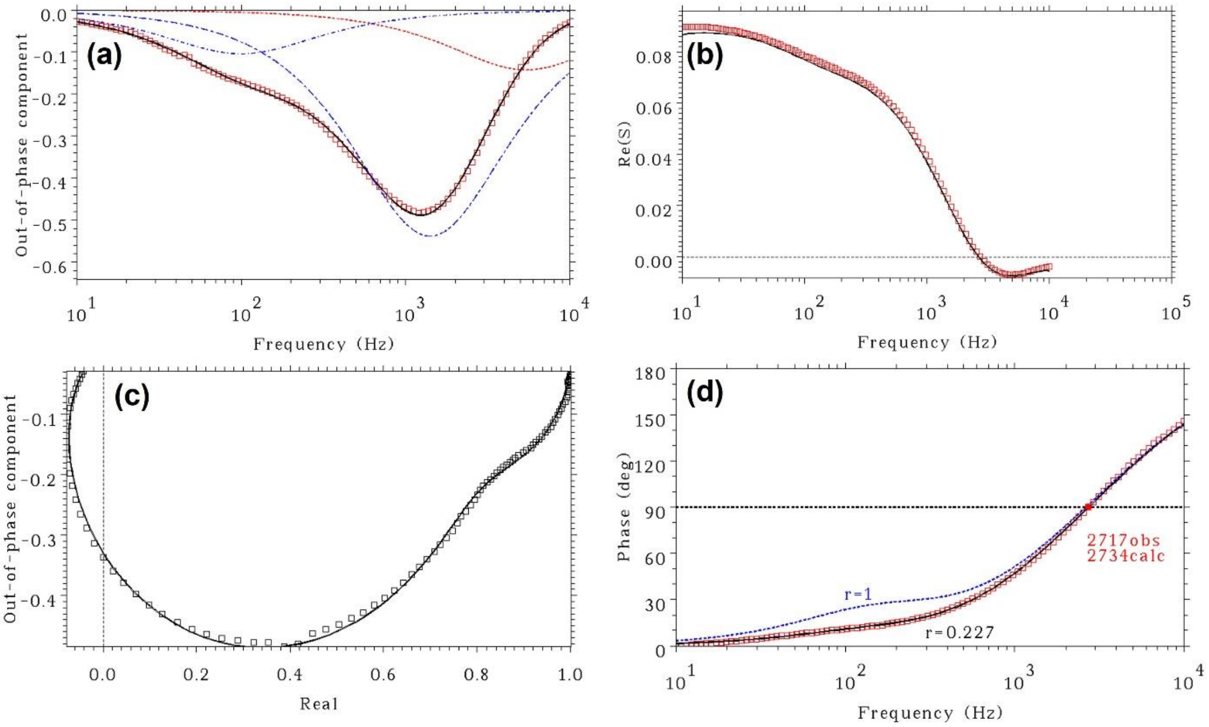

Figure S3. Frequency-domain characterization of the 543 emission of $NaYF_4$:Yb,Er upconversion nanoparticles in H_2O under sinusoidal excitation at 975 nm with the average excitation intensity of 110 W/cm^2 and modulation degree μ =0.1. **(a)** Quadrature data fitting (red squares for experimental data, black solid line for fitting to Eq. (16), and dashed lines for different basis components with the red dashed line having a positive sign). **(b)** Kramers-Kronig transform of the quadrature data to in-phase data (red squares for experimental in-phase data, black solid line calculated from experimental quadrature data). **(c)** Argand diagram of data (black squares) and fitting (black solid line). The fitting was done simultaneously to Eqs. (15) and (16). **(d)** Phase lag data fitting to Eq. (18). Red squares are experimental data corresponding to the inverse tangent of the ratio of quadrature and in-phase signals, the black solid line for

fitted data of App I, and blue dashed line for fitted data of App II. $r = 1$ represents App II, $r = 0.227$ for App I. Notations 2717 and 2734 refer to the experimental and calculated frequencies where the phase lag is 90°.

REFERENCES

1. Liu, H.; Xu, C. T.; Lindgren, D.; Xie, H.; Thomas, D.; Gundlach, C.; Andersson-Engels, S., Balancing power density based quantum yield characterization of upconverting nanoparticles for arbitrary excitation intensities. *Nanoscale* **2013,** *5* (11), 4770-4775.
2. Straume, M.; Frasier-Cadoret, S. G.; Johnson, M. L., Least-Squares Analysis of Fluorescence Data. In *Topics in Fluorescence Spectroscopy: Principles*, Lakowicz, J. R., Ed. Springer US: Boston, MA, 1991; Vol. 2, pp 177-240.
3. Lakowicz, J. R., Frequency-Domain Lifetime Measurements. In *Principles of Fluorescence Spectroscopy (Third Edition)*, Lakowicz, J. R., Ed. Springer US: 2006; pp 157-204.
4. Kissick, D. J.; Muir, R. D.; Simpson, G. J., Statistical Treatment of Photon/Electron Counting: Extending the Linear Dynamic Range from the Dark Count Rate to Saturation. *Anal. Chem.* **2010,** *82* (24), 10129-10134.
5. Press, W. H.; Teukolsky, S. A.; Vetterling, W. T.; Flannery, B. P., *Numerical Recipes in FORTRAN: The Art of Scientific Computing*. 2nd ed.; Cambridge University Press: 1992.
6. Chandler, J. P. *STEPIT, QCPE distribution no. 307, Quantum Chemistry Program Exchange, Program 66, Chemistry Department, Indiana University*, 1971.
7. NIST/SEMATECH e-Handbook of Statistical Methods. https://www.itl.nist.gov/div898/handbook/pmd/section4/pmd444.htm.
8. Hyppänen, I.; Soukka, T.; Kankare, J., Frequency-domain measurement of luminescent lanthanide chelates. *The Journal of Physical Chemistry A* **2010,** *114* (30), 7856-7867.
9. Riuttamäki, T.; Hyppänen, I.; Kankare, J.; Soukka, T., Decrease in luminescence lifetime indicating nonradiative energy transfer from upconverting phosphors to fluorescent acceptors in aqueous suspensions. *The Journal of Physical Chemistry C* **2011,** *115* (36), 17736-17742.
10. Kankare, J.; Hyppänen, I. Frequency-domain measurements. *In Lanthanide Luminescence,* Springer, **2010**; pp 279-312.
